# A novel method for cross-species gene expression analysis

**DOI:** 10.1186/1471-2105-14-70

**Published:** 2013-02-27

**Authors:** Erik Kristiansson, Tobias Österlund, Lina Gunnarsson, Gabriella Arne, D G Joakim Larsson, Olle Nerman

**Affiliations:** 1 Department of Mathematical Statistics, Chalmers University of Technology/University of Gothenburg, Gothenburg, Sweden; 2 Department of Chemical and Biological Engineering, Chalmers University of Technology, Gothenburg, Sweden; 3 Institute of Neuroscience and Physiology, the Sahlgrenska Academy at the University of Gothenburg, Gothenburg, Sweden; 4 Sahlgrenska Cancer Center, Department of Pathology, Sahlgrenska Academy at The University of Gothenburg, Gothenburg, Sweden; 5 Department of Infectious Diseases, Institute of Biomedicine, The Sahlgrenska Academy at the University of Gothenburg, Gothenburg, Sweden

**Keywords:** Gene expression, Evolution, Meta-analysis, Orthologs, Paralogs, Microarray, RNA-seq

## Abstract

**Background:**

Analysis of gene expression from different species is a powerful way to identify evolutionarily conserved transcriptional responses. However, due to evolutionary events such as gene duplication, there is no one-to-one correspondence between genes from different species which makes comparison of their expression profiles complex.

**Results:**

In this paper we describe a new method for cross-species meta-analysis of gene expression. The method takes the homology structure between compared species into account and can therefore compare expression data from genes with any number of orthologs and paralogs. A simulation study shows that the proposed method results in a substantial increase in statistical power compared to previously suggested procedures. As a proof of concept, we analyzed microarray data from heat stress experiments performed in eight species and identified several well-known evolutionarily conserved transcriptional responses. The method was also applied to gene expression profiles from five studies of estrogen exposed fish and both known and potentially novel responses were identified.

**Conclusions:**

The method described in this paper will further increase the potential and reliability of meta-analysis of gene expression profiles from evolutionarily distant species. The method has been implemented in R and is freely available at http://bioinformatics.math.chalmers.se/Xspecies/.

## Background

Gene expression microarray and RNA-seq provide fast and cost-efficient measurement of mRNA abundance for thousands of genes simultaneously. The amount of gene expression data generated by these techniques is constantly increasing and public repositories such as Gene Expression Omnibus and ArrayExpress contains today a large body of information from a wide range of species and experimental conditions [1,2]. Large-scale gene expression assays are however plagued with high variability which complicates data interpretation. The abundance of mRNA is stochastic by nature, both on a cellular and multicellular level [3,4], and there are often large variability between gene expression patterns from different organisms [5]. In addition, technical parameters such as tissue heterogeneity, probe affinities and batch effects may introduce substantial levels of noise [6-8]. Gene expression data is therefore non-trivial to analyze and to put into a biological context.

One way to increase the potential of large-scale gene expression analysis is to combine information between different species. If a biological process is evolutionarily conserved between two species, it is also likely that the transcriptional responses associated with that process share similarities. Indeed, cross-species meta-analysis of gene expression profiles has previously been used to address many questions in biology and medicine. For example, gene expression analysis performed in model species such as mouse and rat are commonly used to study human diseases [9] including cancer [10,11], Alzheimer’s disease [12], diabetes [13] and hypertension [14]. Comparative analysis of gene expression profiles in human and mouse embryonic stem cells has been used to identify similarities and differences associated with the developmental biology in these species [15]. Cross-species meta-analysis has also proven useful in biogeronotology where evolutionarily conserved age-related gene expression responses have been identified based on data from several species, including the fruit fly *Drosophila melanogaster* and the worm *Caenorhabditis elegans*[16,17]. Another example is ecotoxicology, where changes of molecular biomarkers are used to detect toxic effects and to monitor populations and ecosystem health [18]. Such biomarkers should be as general as possible and thus responsive in a wide range of species. Meta-analysis of gene expression profiles from multiple species therefore provides a powerful tool for identification and evaluation of biomarkers [19,20].

Cross-species meta-analysis is however not straight-forward. Different species have different genomes and thus also essential differences in their transcriptomes. The evolutionary process of the eukaryotic genome includes events such as duplication and recombination, which creates complex relations between genes [21]. There is no guarantee that genes from different species with a shared common ancestry (orthologs) have a one-to-one correspondence since gene duplications after speciation may have resulted in one or more additional gene copies (in-paralogs). For species with a relatively short evolutionary distance, such as human and mouse, the number of in-paralogs is low (5.9% of all homologs according to Homologene release 65). The numbers are however higher for species with larger evolutionary distance. For example, 9.6% of all human homologs in *Drosophila melanogaster* have at least one in-paralog and the corresponding numbers for *Saccharomyces cerevisiae* and *Arabidopsis thaliana* are 13.2% and 51% respectively (Homologene release 65). The function of paralogous genes tends to diverge over time and have in general a high gene expression diversity compared to single-copy genes [22-27]. Hence, information from all genes, including both orthologs and paralogs, is vital for cross-species analysis of gene expression profiles.

Several methods have previously been suggested for cross-species analysis of gene expression profiles. Fisher’s combined probability test, which transforms p-values from any number of tests into one single p-value, has been a popular method for comparing multiple gene expression experiments [28-31]. Another approach, which was developed by Stuart et al., was used to compare gene expression of homologs (identified using reciprocal best BLAST hits) over a wide range of experimental conditions [32]. Le *et al.* developed a computationally efficient procedure that compares the distance between ranks of genes from pairs of species [33]. The method was then applied to a large set of microarrays from man and mouse. Another method called mDEDS was developed by Campain and Yang and uses several different statistical measures to perform cross-species comparison of gene expression profiles [30]. Other methods includes LOLA [34] and L2L [35] which are both online tools for comparisons of ranking lists of differentially expressed genes from microarrays studies, including lists from different species. However, all these methods assume a one-to-one correspondence between genes from different species. This assumption may be acceptable when comparing relatively closely related species such as mouse and man, but it makes these procedures inapplicable when comparing more distantly related species.

Lu and co-authors have previously developed methods for analysis of gene expression between different species that takes many-to-many relations into account [36-38]. By using Markov random fields and belief propagation, they were able to identify cell cycling genes in human and yeast [37]. The methods were also used to analyze genes which shared expression profiles in human and mice infected by various pathogens [38]. However, the topology of the Markov random fields depends on the experimental design which makes them hard to adapt to many forms of gene expression experiments. They also make explicit assumption of the distribution of the gene expression, either in the form of an extreme value distribution [37] or a Gaussian distribution [38]. This makes them unsuitable for many heterogeneous datasets with observations from multiple measurement platforms, such as gene expression microarrays and RNA-seq. To enable cross-species meta-analysis of existing and future gene expression data, novel flexible methods that can handle many-to-many relationships between genes are needed [30,39].

In this paper we describe a new statistical method for meta-analysis of gene expression profiles from different species. The method was derived to take all orthologous and co-orthologous genes into account. Similar to Fisher’s method, the proposed method uses gene-specific p-values, which makes it applicable to many forms of measurement platforms including microarrays and sequencing based techniques such as RNA-seq. A simulation study showed that the proposed method resulted in a substantial gain of statistical power for identification of differentially expressed genes. As a proof of concept, we used the method to identify evolutionarily conserved regulation of stress responsive genes in eight species subjected to heat stress. We also applied the method to gene expression data from aquatic vertebrates exposed to estrogens to demonstrate its applicability within ecotoxicology.

## Results

### A novel method for cross-species analysis of gene expression

Assume that a number of large-scale gene expression experiments have been performed in a set of species investigating an evolutionarily conserved transcriptional response. Assume further that each experiment has been analyzed individually resulting in a p-value for each measured gene describing the significance of the differential expression (e.g. between two treatments). We will also assume that there is a fixed and known evolutionary structure describing all groups of orthologous and co-orthologous genes present in the species of interest. Such homology groups are readily available from multiple sources, such as Homologene [40], OrthMCL-DB [41] and InParanoid [42] or can alternatively be inferred *de novo* by tools such as OrthoMCL [43].

The method proposed in this paper operates on the gene-specific p-values generated from each experiment. For each homology group and species, the method summarizes all in-paralogs into one single value by selecting the minimum (most significant) p-value. A weighted score is then calculated by summing the negative logarithms of the minimum p-values from each gene expression experiment. A combined p-value for each homology group is finally derived by comparing the observed score to the null distribution which has a known, but non-trivial, analytic form. Finally, a Benjamini-Hochberg false discovery rate (FDR) is calculated to control for the multiple testing of several homology groups (typically ∼10,000 homology groups are tested).

The weights used to combine the different experiments are based on the evolutionary structure. Under the assumption of no differential expression, genes with many in-paralogs are more likely to result in a lower minimum p-value than genes with few or no in-paralogs. The weights therefore decrease with the number of in-paralogs to generate an unbiased score. The weights also contain an arbitrary component, which can be used to weigh individual experiments up or down. For example, the arbitrary weights can be used to prevent bias if multiple experiments are performed in the same species.

Full mathematical details, including the derivation of the weights and the analytical null distribution, can be found in Methods. An R-implementation of the methods if freely available at http://bioinformatics.math.chalmers.se/Xspecies/.

### Evaluation of the statistical power

The statistical power of the proposed method was investigated using simulations together with three other procedures that have been previously suggested for handling of in-paralogous genes. The following four approaches were analyzed 

(i) The proposed method: the most significant p-value of the in-paralogs in each species is combined across species.

(ii) The combination method: the expression data from in-paralogs are treated as independent biological replicates from the same gene [44].

(iii) The average method: expression data from in-paralogs are combined into one single observation by taking the average value of the raw expression data [39].

(iv) The random method: only expression data from one in-paralog is used (randomly selected). All other values are discarded [39].

For the combined, average and random method the cross-species p-value is calculated by Fisher’s combined probability test.

**Figure 1:**
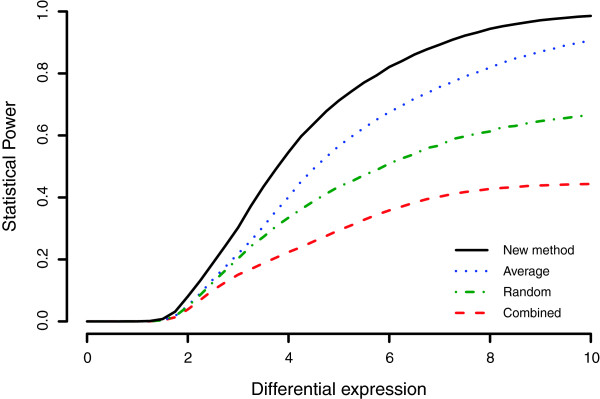
**Comparison of the statistical power.** The proposed method (solid black) results in a substantial increase in power of detecting homology groups that were differentially expressed in multiple species compared to other methods (*average* - dotted blue, *random* - mixed green and *combined* - dashed red). The x-axis shows the size of the differential expression and the y-axis the corresponding power. See Methods for full details about the simulation.

Homology groups from eight different species containing at least two in-paralogs in at least one of the species were used in the simulations (the same species as used in the heat stress data analysis below). The simulations were performed in the simplest possible setting where data corresponding to two treatment groups was generated for each experiment by sampling the Gaussian distribution (*μ*=0,*σ*^2^=1). Ten percent of the homology groups was randomly selected to be differentially expressed and for each such group an effect ranging from 0 to 10 was added to one single in-paralog (x-axis of Figure 1). P-values were calculated based on the two-population t-test assuming equal variances (see Methods for full details).

**Figure 2:**
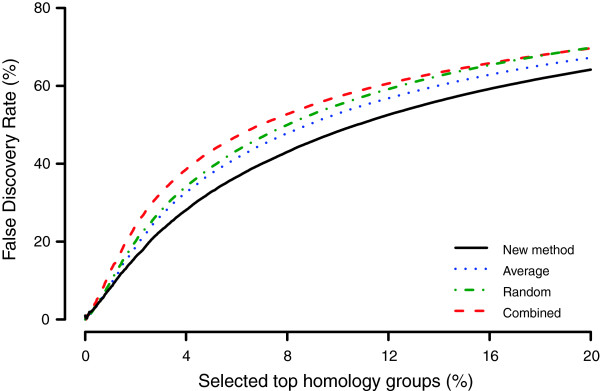
**Comparison of the false discovery rate.** The false discovery rate (FDR) decrease when homology groups were ranked with the proposed method (solid black) compared to other methods (*average* - dotted blue, *random* - mixed green and *combined* - dashed red). The FDR was simulated for differentially expressed genes with a small effect (*μ* = 2, *σ*^2^ = 1). The x-axis shows percentage of selected genes and the y-axis the true false discovery rate. See Methods for full details about the simulation.

Figure 1 shows the power as a function of the size of the differential expression. The proposed method had a substantially higher power than other approaches among which the average method performed best followed by random and combined methods. The increased statistical power had a high impact on the false discovery rate, which was considerable lower for the proposed method. At a relatively small effect of *μ* = 2, the false discovery rate among the 5% most significant groups was 32.8% for the proposed method and 37.5%, 39.1% and 43.2% for the average, random and combined methods (Figure 2). The corresponding numbers of the false discovery rate for *μ* = 5 were 0.64%, 1.1%, 2.9%, 7.1% for the proposed, average, random and combined methods respectively.

The methods were also evaluated using simulations in more diverse settings. When a second in-paralog was differentially expressed in the same direction, i.e. the same effect added to two genes, the performance of the combined and average method increased (Additional file 1: Figure A1). However, when an effect in the opposite direction was added to a second in-paralog (half of the effect subtracted), the power of the average method decreased substantially. At an effect of 6, the power of the average method was reduced from 0.68 to 0.28 while the power for the proposed method decreased from 0.82 to 0.71 (Figure 1 and Additional file 1: Figure A2). When the normal distribution was replaced by a t-distribution with five degrees of freedom, the power decreased equally for all methods (Additional file 1: Figure A3). A similar result was seen when errors were introduced in the homology structure by randomly replacing orthologous genes with non-orthologous genes from the same species (Additional file 1: Figure A4 and A5).

### Evolutionarily conserved expression changes in response to heat stress

The cellular response to heat stress is comprised by multiple mechanisms that protect the cell from damage. One of the most vital parts of this defense system is the molecular chaperons which stabilizes and folds proteins into their proper conformations. Chaperons are present in all living organisms and their gene expression response, which is known to be evolutionarily conserved, has been studied in detail [45,46]. To test the model proposed in the study in a biological context, we analyzed gene expression data from heat stress experiments performed in eight species ranging from yeast to man (Table 1).

**Table 1:** A summary of the experiments used in the meta-analysis of heat stress

**Organism**	**Samples**	**Temperature**	**Treatment length**	**Reference**
*Homo sapiens*	3+3	42°C	1 h	GEO:GSE7458, [47]
*Mus musculus*	3+3	42°C	40 min	GEO:GSE14869, [48]
*Danio rerio*	3+3	37°C	1 h	GEO:GSE17949 (unpublished)
*Drosophila melanogaster*	2+4	36°C	1 h	GEO:GSE5147, [49]
*Oryza sativa*	3+3	42°C	3 h	GEO:GSE14275, [50]
*Arabidopsis thaliana*	4+4	38°C	1 h	[51]
*Schizosaccharomyces pombe*	2+4	39°C	1 h	ArrayExpress:E-MEXP-29, [52]
*Saccharomyces cerevisieae*	5+5	37°C	15 min	GEO:GSE8335, [53]

**Figure 3:**
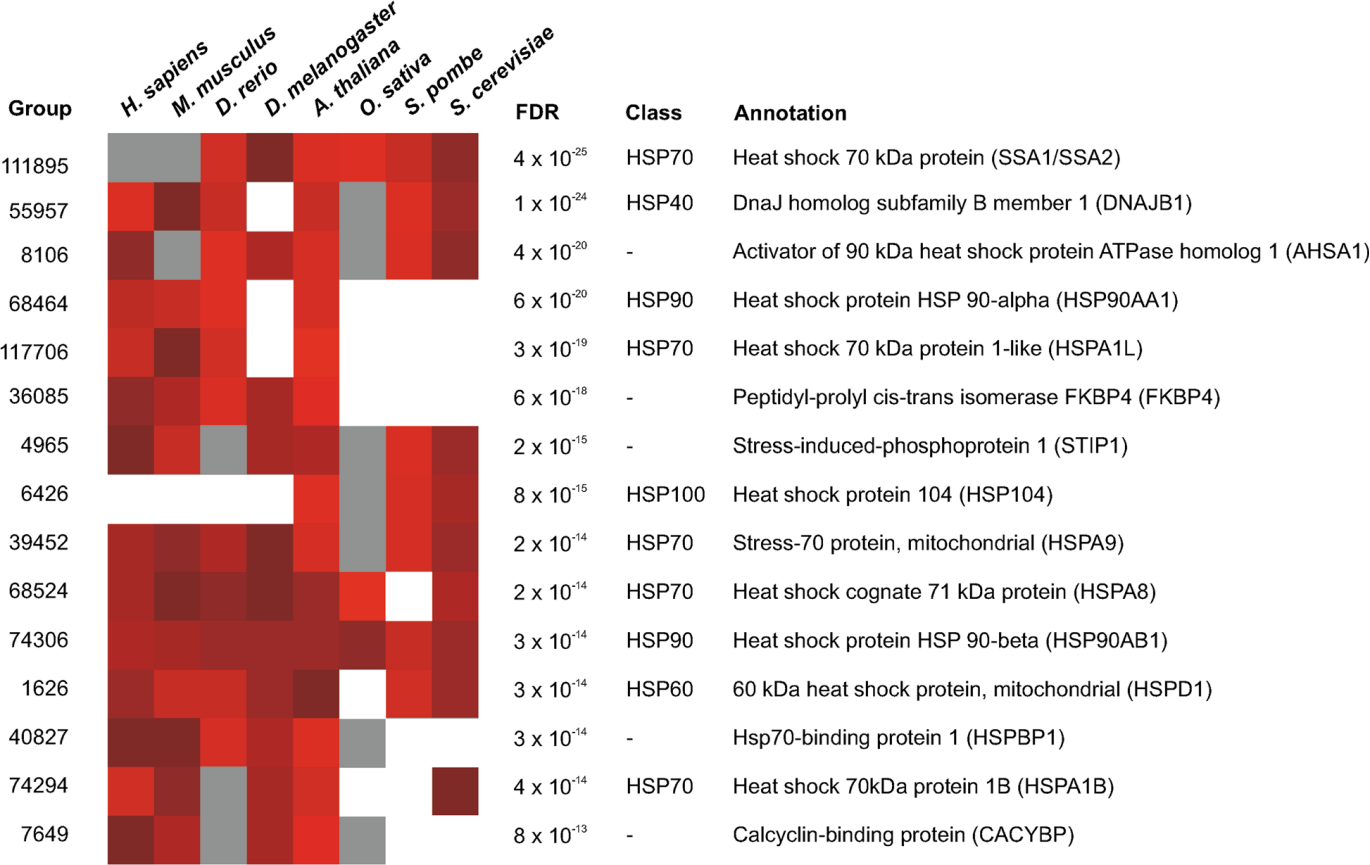
**The most significant homology groups in the cross-species analysis of heat stress.** The figure shows the 15 most significant significant homology groups from cross-species analysis of heat stress microarray data with homologs in at least four of the eight species. All of the 15 homology groups were up-regulated during heat stress. The heatmap shows the contribution from each individual experiment where higher intensity corresponds to a more significant p-value. White squares indicate the absence of a homologous gene while grey squares indicate the presence of homologs that have not been measured (e.g. missing one the microarray). The other columns in the figure corresponds to the Homologene accession number (Group), the false discovery rate (FDR), the chaperon class (Class) and a gene description (Annotation). Full results for all 37909 homology groups are available as Additional file 2.

The NCBI Homologene release 65 database was used to retrieve 37909 homology groups connecting the genes from the eight species. Of these were 28241 (74.5%) represented by at least one observation in at least one experiment. Among the represented groups, 11049 (39.1%) had at least one in-paralog in at least one species and the traditional Fisher’s method was thus not applicable to this dataset. Applying the method proposed in this paper resulted in 1074 homolog groups (3.8%) with a false discovery rate less than 0.01. In contrast, the combined, average and random methods resulted in 552, 795 and 586 groups with an FDR less than 0.01 respectively (Additional file 2). Among the 15 most significant homology groups identified by the proposed method (Figure 3), ten were molecular chaperons corresponding to four of the five major chaperon super families (Hsp60, Hsp70 Hsp90, Hsp100) [45]. The fifth family, the small heat stress proteins (sHSP), is less well-conserved and thus less clustered, was still found significant in smaller homology groups (e.g. homology group 93388 with genes from *A.thaliana* and *O. sativa*, FDR = 2.7×10^−9^). The most significant homology groups (FDR≤0.01) were tested for functional enrichment of Gene Ontology terms. Not surprisingly, many of the significant terms were associated with heat stress, including response to stress (GO:0006950, *p* = 1.5 × 10^−27^), response to temperature stimulus (GO:0009266, *p* = 8.7×10^−15^) and protein refolding (GO:0042026, *p* = 1.0×10^−10^). We also observed that GO terms associated with other biological functions and processes, such as processes involving non-coding RNA (e.g. GO:0030515 snoRNA binding, GO:0034660 ncRNA metabolic process) and ribosome synthesis (e.g. GO:0042254) were significant. See Additional files 2 and 3 for full results.

**Figure 4:**
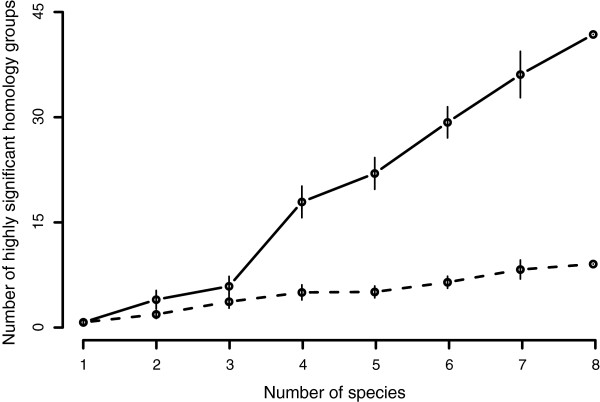
**Highly significant heat stress homology groups.** The number of highly significant (*p* < 10^−6^) differentially expressed homology groups regulated by heat stress (y-axis) increased when more species were included in the analysis (x-axis). The figure was created by performing a cross-species analysis for all possible configurations containing *n* species (with 1 ≤ *n* ≤ 8). For each fixed value of *n*, the average number of highly significant p-values were calculated. The error bars shows the corresponding standard deviations. The dashed curve was calculated by performing the same analysis on homology groups with randomized homology groups (the sizes of the homology groups were fixed).

The analysis of the heat stress data also revealed that the number of highly significant genes (unadjusted *p* < 10^−6^) increased with the number of included experiments. When each dataset were analyzed individually, only two of the eight experiments resulted in genes with p-values less than 10^−6^ (two and three genes in the datasets from *A. thaliana* and *O. sativa* respectively). As more species were combined, the number increases monotonously (Figure 4, solid line) and when all eight experiments were included, 42 homology groups had a p-value less than 10^−6^. The effect was reduced when the evolutionary relationships between genes from different species were removed by randomization of the homology groups (Figure 4, dashed line).

### Analysis of the transcriptional responses to estrogens in fish

**Figure 5:**
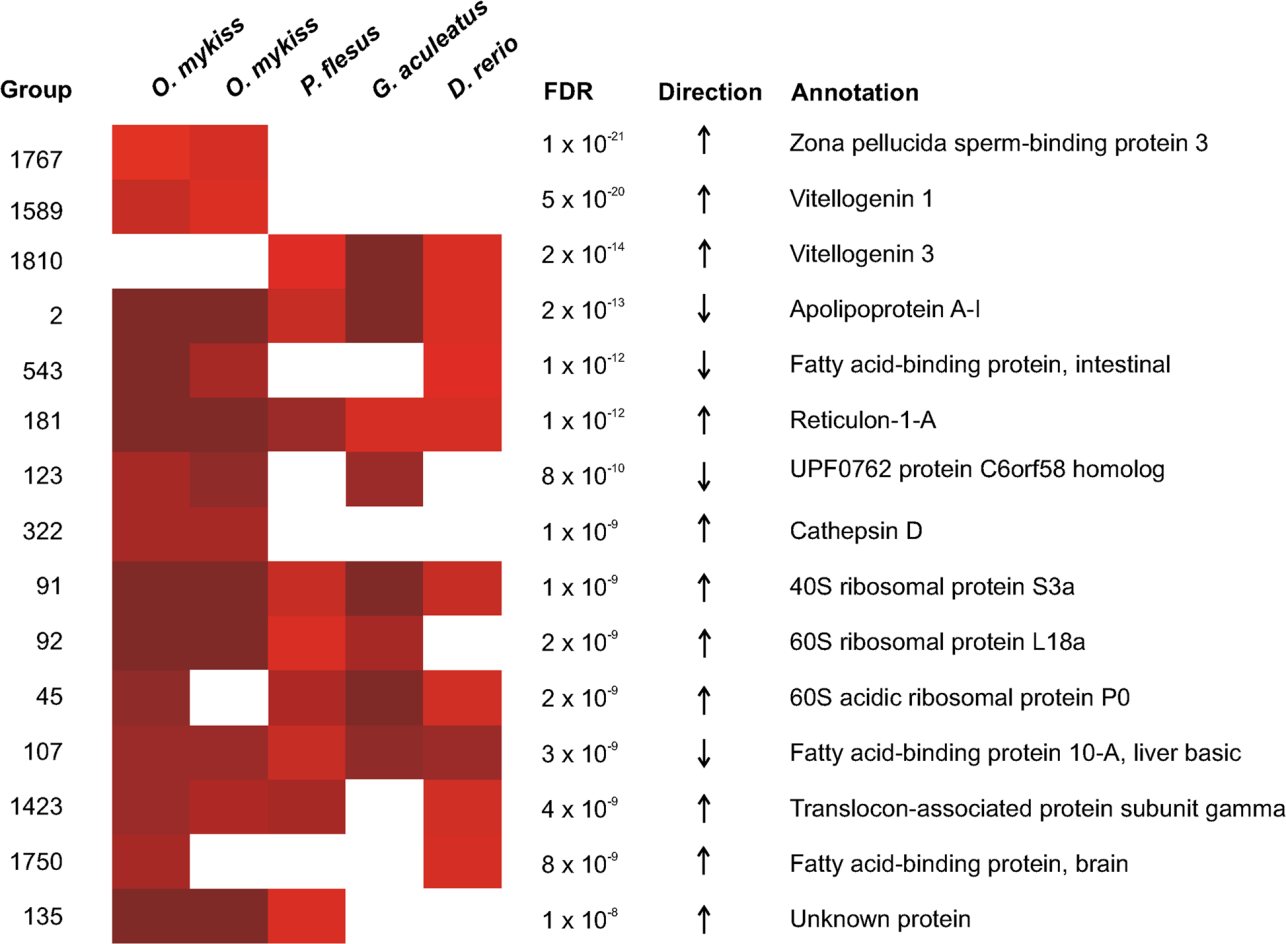
**Cross-species analysis of fish exposed to estrogens.** The figure shows the 15 most significant homology groups from five microarray studies with estrogenic exposed fish. Only homology groups with gene expression data from at least two of the five studies are shown. The heatmap describes the contribution from each individual experiment where higher intensity corresponds to a more significant p-value. White squares indicate the absence of a p-value (e.g. no homlogous gene or gene missing on the microarray). The columns in the figure corresponds homology group identifier (Group), the false discovery rate (FDR), the direction of the differentially expression (Direction) and a gene description (Annotation). Full results for all 6449 homology groups are available as Additional file 4.

Estrogenic substances reach the aquatic environment, for example via municipal waste water, and can affect the reproductive health of wild fish [54-57]. To investigate the evolutionarily conserved transcriptional response to estrogen exposure we applied the method to data from five microarray studies on hepatic gene expression data from juvenile or male fish (Table 2). OrthoMCL was used to identify 5640 homology groups containing genes included on the microarrays. Among these groups, 4701 (83.4*%*) had at least one in-paralog in at least one species. Analysis with the proposed method resulted in 549 homology groups with a false discovery rate less than 0.01 of which 430 had homologs in at least two species. The 15 most significant homology groups (Figure 5) contained many well-established estrogen responsive genes, such as zona pellucida sperm-binding protein 3, vitellogenin 1, vitellogenin 3 and cathepsin D [58-60]. Among the 15 most significant groups, at least seven have shown to be differentially expressed also on protein level [61,62] and 80% (12) have previously been associated with estrogen exposure in vertebrates according to the Comparative Toxicogenomics Database [63]. Full lists are available as Additional file 4.

**Table 2:** A summary of the experiments used in the meta-analysis of estrogen-exposed fish

**Organism**	**Samples**	**Exposure**	**Exposure length**	**Reference**
*Platichthys flesus*	5+5	E_2_, injected, 10 mg/kg	8 days	Pers. com. TD Williams, [64]
*Gasterosteus aculeatus*	3+3	E_2_, water, 50 ng/L	2 days	Pers. com. TD Williams, [65]
*Danio rerio*	4+4	EE_2_, water, 10 ng/L	21 days	GEO:GSE7220, GEO: [66]
*Oncorhynchus mykiss*	2+2	E_2_, dietary, 5ppm	12 days	GEO:GSE7837, [67]
*Oncorhynchus mykiss*	4+4	EE_2_, water, 10 ng/L	14 days	ArrayExpress:E-MEXP-1149, [19]

Furthermore, several significant homology groups contained genes that were not identified as estrogen responsive by any of the individual studies, e.g. fatty acid desaturase 2 (group 582, FDR= 1.5×10^−7^), sodium/potassium-transporting ATPase subunit alpha-1 (group 61, FDR= 7.8×10^−6^) and translocon-associated proteins delta and gamma (groups 561 and 1423, FDR= 2.9×10^−8^ and 3.9×10^−9^ respectively). These genes have all previously been shown to be estrogen responsive in mammals [68-70]. In addition, the translocon-associated protein subunit delta has been shown to be differentially expressed on protein level in *Danio rerio* exposed to estrogen [61].

## Discussion

Meta-analysis of gene expression profiles is hampered by the lack of a one-to-one correspondence between orthologous genes from different species. Evolutionary events, such as gene duplications, have resulted in paralogous genes which makes traditional approaches for meta-analysis inapplicable. We therefore developed a new statistical method for meta-analysis of gene expression profiles between experiments performed in evolutionarily distant species. The method takes advantage of the homology structure between the species of interest and can therefore take any number of orthologous and co-orthologous genes into account. The method is general in the sense that it operates on p-values from individual gene expression experiments and is therefore independent of the type of the raw gene expression data. This makes the method applicable to any gene expression measurement platform, including DNA microarrays and quantitative PCR as well as techniques based on sequencing such as RNA-seq. Using p-values also makes it possible to include results from already analyzed experiments where the raw data is not publicly available or missing.

The proposed method can be seen as an extension of Fisher’s combined probability test [28], which is widely used statistical method for meta-analysis. In fact, when no in-paralogous genes are present in any of the species, the proposed method and Fisher’s method are equivalent. Similarly to the Fisher’s combined probability test, the proposed method is dependent on the validity of the statistical models used to analyze the individual experiments. The combined cross-species p-values are calculated from an analytical distribution derived based on the assumption of gene-specific p-values that are independently and uniformly distributed under the null hypothesis. An alternative approach, which is less dependent on the model assumptions, is to use permutations [71]. For many experimental designs, the null-distribution can be estimated by randomly permuting the labels of the samples in each experiment. However, permutation-based estimation of the null-distribution requires a relatively large number of biological replicates in order to generate a sufficiently large number of permutations. The heat stress data analyzed in this study had, for example, too few observations for estimation of the null-distribution using permutations.

Cross-species meta-analysis of gene expression is dependent of the evolutionary relationship between the orthologous and co-orthologous genes present in the species of interest. Identification of homologous genes in evolutionarily distant species is however complex and can result in false predictions [72]. Such errors will either group non-related genes in the same homology group or, vice versa, scatter homologous genes between different homology groups. Since the proposed method assumes that the evolutionary structure is known and correct, such errors will affect the results negatively. Improved and more accurate algorithms for predicting homologous genes will thus further increase the potential of cross-species meta-analysis of gene expression. On the other hand, the conserved expression profiles generated by the proposed method can be used to correct false predictions of homology. In the heat stress analysis Homologene group 111895 (HSP70-homologs, Homologene release 65) was found to be highly significant in all species except for *D. melanogaster*. Interestingly, a closer examination of that homology group showed that the HSP70 functional domain was missing from the *D. melanogaster* gene and which suggests that it may indeed not be a true homolog.

The statistical power of the proposed method and three previously suggested methods for combining multiple observations in microarray analysis was evaluated using simulations. The proposed method was the only solution that was explicitly developed to handle in-paralogous genes and its power was, not surprisingly, considerably higher (Figure 1). The resulting false discovery rate was also lower (Figure 2). When multiple in-paralogs from the same homology group had a similar transcriptional pattern the difference in performance between the methods was reduced. However, when then multiple in-paralogs showed a divergent transcriptional pattern, the difference in performance increased in favor of the proposed method. This reflects the underlying assumptions, where the proposed method assumes that only one of the in-paralogs in homology group is differentially expressed while the others are non-responsive. The combination and average methods does, on the other hand, assume that all in-paralogs are affected by the treatment. It should also be noted that conditions used in the simulations are idealized and the results should therefore be interpreted as such. Real gene expression data does not follow a Gaussian distribution and has a complex correlation structure, both between genes and samples [6,73-75]. The simulation study shows, however, that the loss in statistical power of detecting differentially expressed genes in cross-species meta-analysis may be substantial if in-paralogs are not properly incorporated in the analysis.

The proposed method was used to compare the gene expression response to heat stress based on microarray data from eight eukaryotes. The analysis identified several well-known mechanisms involved in the transcriptional response to heat. Most pronounced was the up-regulation of molecular chaperons and 10 of the 15 most significant homology groups corresponded to heat stress proteins from four of the five major chaperon families (Figure 3). Functional enrichment of gene ontology terms revealed additional biological processes associated with the cellular response to heat. The number of significant homology groups was also shown to increase with the number of included species. These results show that the proposed model generated biologically relevant results by combining gene expression profiles from evolutionarily distant species. Analysis of evolutionarily conserved gene expression changes under heat stress has previously been suggested as an efficient approach to further understand the underlying biological processes [45]. It is therefore plausible that a more in-depth analysis of our result from the cross-species meta-analysis may result in more insights and novel findings within this area.

Inter-species extrapolations is a cornerstone of ecotoxicological risk assessment since only a tiny fraction of the species present in the environment can be studied in the laboratory [76]. Comparisons of inter-species gene expression profiles provide an attractive way to identify evolutionarily conserved modes of action and novel biomarkers of exposure or effect. We therefore used the proposed method to find common transcriptional responses in four different fish species. The analysis revealed several known and well-established responses of estrogen, some which have been associated with adverse physiological effects. The method also identified differentially regulated genes that were not classified as estrogen responsive by the individual experiments. This shows that the method can be used to identify evolutionarily conserved transcriptional responses to toxicants in ecologically relevant species and it demonstrates the potential of cross-species meta-analysis within ecotoxicology.

Cross-species analysis of gene expression is dependent on the similarities in the transcriptional responses of the studied species. However, evolutionarily distant species have fundamental differences in their physiology which makes it hard, or even impossible, to perform experiments under identical conditions. Even though the associated biological processes are evolutionarily conserved the differences in experimental design and execution can introduce substantial variability in the transcriptional responses. In the cross-species analysis of heat stress we included data from eight species that were treated with different degrees of heat stress during different time spans. There were also differences in the designs of the estrogen exposures, e.g. exposure concentrations, times and routes. Our results show, however, that for both these examples of cross-species analysis, the experiments were similar enough to generate biological relevant results. It is, on the other hand, hard to estimate what evolutionarily conserved transcriptional responses that are not identified due to differences in the experimental designs.

## Conclusion

Cross-species analysis of gene expression is complicated by the non-trivial relationships between genes from different species. The new statistical method proposed in this study takes the evolutionary structure into account and can therefore compare transcriptional profiles from species with any number of orthologous and co-orthologous genes. The performance of the proposed method, compared to other existing solutions, was therefore considerably higher when in-paralogous genes are present. As a proof-of-concept, the method was used to identify evolutionarily conserved transcriptional responses in microarray data from heat stress experiment performed in eight diverse species. The applicability of the method within ecotoxicology was also demonstrated by the identification of known and novel responses in fish exposed to estrogens. An implementation of the method for the statistical language R is available for free at http://bioinformatics.math.chalmers.se/Xspecies/.

## Methods

### Mathematical details

Assume that we are interested in a meta-analysis of gene expression profiles from *m* experiments performed in *m* species (which does not have to be unique). Assume also that the orthologous and co-orthologous genes ( [21]) of the species are described by *n* homology groups $G_{1},\ldots ,G_{n}$ where each group $G_{i}$ be defined as

$$G_{i}=\left\{G_{i1},\ldots ,G_{\text{im}}\right\}$$

and where *G*_*ij*_ is the set of genes in group $G_{i}$ for species *j*. Assume further that there are *l*_*ij*_ such genes in group *i* and species *j*, i.e.

$$G_{\text{ij}}=\left\{g_{\text{ij}1},\ldots ,g_{\text{ij}l_{\text{ij}}}\right\}.$$

It follows that any pair of genes *g*_*ijk*_ and $g_{ij^{\prime }k^{\prime }}$ in homology group *i* are in-paralogs if *j* = *j*^′^ and orthologs or co-orthologs if *j* ≠ *j*^′^.

Assume that experiments have been performed measuring the gene expression for each gene *g*_*ijk*_ and that differential expression is tested using the hypotheses

$$\begin{array}{ll} H_{\text{ijk}}^{0}: & \text{gene}g_{\text{ijk}}\text{is not differentially expressed},\\ H_{\text{ijk}}^{A}: & \text{gene}g_{\text{ijk}}\text{is differentially expressed}, \end{array}$$

resulting in a p-value *p*_*ijk*_ (only the two-sided hypothesis will be considered, the generalization to one-sided hypotheses is straight forward). The p-values are assumed to follow a similar structure as the homolog groups, i.e.

$$P_{i}=\left\{P_{i1},\ldots ,P_{\text{im}}\right\}\;\text{where}\;P_{\text{ij}}\;\text{is}\;P_{\text{ij}}=\left\{p_{\text{ij}1},\ldots ,p_{\text{ij}l_{\text{ij}}}\right\}.$$

For each homology group *i* we will test

(1) $$\begin{array}{l} H_{i}^{0}:\text{None of the genes in}G_{i}\text{are}\\ \;\text{differentially expressed} \end{array}$$

versus the alternative that $H_{i}^{0}$ is not true. Let ${\tilde{p}}_{\text{ij}}$ be the most significant p-value for paralogs in group *i* and species *j*, i.e.

$${\tilde{p}}_{\text{ij}}=\underset{k=1,\ldots ,l_{\text{ij}}}{\text{min}}p_{\text{ijk}}.$$

The statistic that will be used to test (1) is the cross-species score *S*_*i*_ defined as

$$S_{i}=\overset{m}{\underset{j=1}{\sum }}w_{j}K_{\text{ij}}\log{\tilde{p}}_{\text{ij}}$$

where *K*_*ij*_ is a constant and *w*_*j*_ are arbitrary experiment-specific weights summing to 1.

The null distribution of *S*_*i*_ is non-trivial and will now be derived. Let *X*_*ijk*_ = − log*p*_*ijk*_ and

$$Y_{\text{ij}}=-\log{\tilde{p}}_{\text{ij}}=\underset{k=1,\ldots ,l_{\text{ij}}}{\text{max}}X_{\text{ijk}}$$

Under the assumption that $H_{i}^{0}$ is true all p-values {*p*_*ijk*_} are independent and uniformly distributed between 0 and 1. Hence, *X*_*ijk*_ is exponentially distributed with intensity 1 and *Y*_*ij*_ is the maximum of *l*_*ij*_ such independent exponentially distributed random variables. By rewriting *Y*_*ij*_ as a sum of the order statistic $\left(X_{\text{ij}(1)},\ldots ,X_{\text{ij}(l_{\text{ij}})}\right)$ of *X*_*ij*1_,…,*X*_*ijk*_, i.e.

$$\begin{array}{l} Y_{\text{ij}}=\max\left(X_{\text{ij}1},\ldots ,X_{\text{ijk}}\right)=X_{\text{ij}(1)}+\\ \;+(X_{\text{ij}(2)}-X_{\text{ij}(1)})+\ldots +(X_{\text{ij}(l_{\text{ij}})}-X_{\text{ij}(l_{\text{ij}}-1)}). \end{array}$$

It follows by the memoryless property of the exponential distribution that *X*_*ij*(1)_∼Exp(1/*n*) and that

() $$\begin{array}{ll} \;\mathbb{P}\left(X_{\text{ij}(2)}\;-\;X_{\text{ij}(1)}\leq x\right) & =\int_{0}^{\infty }\mathbb{P}\left(X_{\text{ij}(2)}\leq x\right)\mathbb{P}\left(X_{\text{ij}(1)}=y\right)\text{dy}\\ & =\mathbb{P}\left(\underset{k=2,\ldots ,l_{\text{ij}}}{\text{min}}X_{\text{ijk}}\leq x\right). \end{array}$$

Thus, *X*_*ij*(2)_−*X*_*ij*(1)_∼Exp(1/(*n*−1)) and by repeating the same arguments *Y*_*ij*_ can be written as

$$Y_{\text{ij}}=\overset{l_{\text{ij}}}{\underset{k=1}{\sum }}\frac{1}{k}Z_{\text{ijk}}$$

where $Z_{\text{ij}1},\ldots ,Z_{\text{ij}l_{\text{ij}}}$ are *l*_*ij*_ independent exponentially distributed random variables with intensity 1. The expected value of *Y*_*ij*_ can be calculated to

$$\text{Exp}[Y_{\text{ij}}]=\overset{l_{\text{ij}}}{\underset{k=1}{\sum }}\frac{1}{k}.$$

If we let

$$K_{\text{ij}}={\left(\overset{l_{\text{ij}}}{\underset{k=1}{\sum }}\frac{1}{k}\right)}^{-1},$$

where *K*_*ij*_ = 0 if *l*_*ij*_ = 0, the cross-species statistic *S*_*i*_ can be written as

$$\begin{array}{ll} \;S_{i} & =\overset{m}{\underset{j=1}{\sum }}w_{j}K_{\text{ij}}\log{\tilde{p}}_{\text{ij}}=\overset{m}{\underset{j=1}{\sum }}w_{j}\frac{Y_{\text{ij}}}{\text{Exp}\left[Y_{\text{ij}}\right]}\\ & =\overset{m}{\underset{j=1}{\sum }}\overset{l_{\text{ij}}}{\underset{k=1}{\sum }}\frac{w_{j}}{k\text{Exp}[Y_{\text{ij}}]}Z_{\text{ijk}}=\overset{m}{\underset{j=1}{\sum }}\overset{l_{\text{ij}}}{\underset{k=1}{\sum }}{\tilde{w}}_{\text{ijk}}Z_{\text{ijk}} \end{array}$$

where the weights ${\tilde{w}}_{\text{ijk}}$ are defined as

$${\tilde{w}}_{\text{ijk}}=\frac{w_{j}}{k\;\text{Exp}[Y_{\text{ij}}]}.$$

*S*_*i*_ is thus a weighted sum of independent exponentially distributed random variables with intensity 1. The weights ${\tilde{w}}_{\text{ijk}}$ contains two parts, an experimental specific weight *w*_*j*_ and 1/(*k*Exp[*Y*_*ij*_]). The latter compensates for the number of paralogs in order to avoid bias from large homology groups. The weights *w*_*j*_ are arbitrarily and can be set to weigh individual experiments up and down. This is for example useful when multiple experiments are performed in a single organism (see Estrogen exposure below for an example). However, more sophisticated weighting strategies are also possible, such as weights based on the evolutionary distance between the included species (e.g. evolutionary distinctiveness score [77]).

The density function of *S*_*i*_ can be calculated explicitly depending on the weights ${\tilde{w}}_{\text{ijk}}$. For the case when all ${\tilde{w}}_{\text{ijk}}$ are different the density function becomes [78]

$$f_{S_{i}}(s)=\overset{m}{\underset{j=1}{\sum }}\overset{l_{\text{ij}}}{\underset{k=1}{\sum }}\frac{{\tilde{w}}_{\text{ijk}}^{ml_{\text{ij}}-2}}{\overset{m}{\underset{j^{\prime }=1,j^{\prime }\neq j}{\prod }}\overset{l_{\text{ij}}}{\underset{k^{\prime }=1,k^{\prime }\neq k}{\prod }}\left({\tilde{w}}_{\text{ijk}}-{\tilde{w}}_{ij^{\prime }k^{\prime }}\right)}e^{-s/{\tilde{w}}_{\text{ijk}}}.$$

Analogously, density functions for the cases when two or more weights are equal can also be derived. However, evaluating the cumulative density function (CDF) requires numerical integration which is computationally expensive. We therefore approximate the distribution of *S*_*i*_ using a Gamma distribution with the same expectation value and variance. Approximating a weighted sum of exponentially distributed variables with a Gamma distribution has previously shown to accurate enough for our purpose [79]. The expected value and variance of *S*_*i*_ becomes

$$\begin{array}{l} \text{Exp}[S_{i}]=1\\ \text{Var}[S_{i}]=\overset{m}{\underset{j=1}{\sum }}w_{j}^{2}\frac{\overset{l_{\text{ij}}}{\underset{k=1}{\sum }}k^{-2}}{{\left[\overset{l_{\text{ij}}}{\underset{k=1}{\sum }}k^{-1}\right]}^{2}} \end{array}$$

Hence, the shape and scale parameters *α* and *β* should be

$$\alpha_{i}=\beta_{i}={\left[\overset{m}{\underset{j=1}{\sum }}w_{j}^{2}\frac{\overset{l_{\text{ij}}}{\underset{k=1}{\sum }}k^{-2}}{{\left[\overset{l_{\text{ij}}}{\underset{k=1}{\sum }}k^{-1}\right]}^{2}}\right]}^{-1}$$

The hypothesis in 1 can now be tested and a corresponding p-value calculated by comparing the observed value *S*_*i*_ with the null distribution of *S*_*i*_.

### Simulations

Simulations were performed on homology groups from Homologene for the species *Saccharomyces cerevisiae* (4932), *Schizosaccharomyces pombe* (4896), *Arabidopsis thaliana* (3702), *Oryza sativa* (4530), *Drosophila melanogaster* (7227), *Danio rerio* (7955) *Mus musculus* (10090), *Homo sapiens* (9606) (NCBI Taxonomy IDs are given in parenthesis). Each gene was assumed to be measured in two different groups, one control and one treated, with three independent observations from each. Data was simulated from a Gaussian distribution with mean value 0 and variance 1 and p-value calculated using a two-population t-test assuming equal variance. For differentially expressed orthologous groups (10%, randomly selected) an effect ranging from 0 to 10 was added to the treated group (e.g. changing the expected value from 0 to the effect). For groups and species with in-paralogous genes the effect was added to one single in-paralog (randomly selected). The weights *w*_*ij*_ in *S*_*i*_ were set to be uniform. For the combined method all observations from in-paralogs treated as independent replicated observations for one single gene (homology group). For the average method, an average was taken over all observations from in-paralogs generating one single observation for each observation. For the random method one of the in-paralogs was randomly selected and other discarded. For these three methods the cross-species p-value was calculated by Fisher’s combined probability test [28]. The false discovery rate for homology group *i* was estimated by calculating the proportion of false positives among the *i* most significant groups.

### Meta-analysis of gene expression

#### Pre-processing and analysis of microarray data

Intensity data from Affymetrix type of microarrays was pre-processed using RMA [80] while intensity data from two-channel microarrays was normalized using global loess [81]. The quality of each microarray was assessed by inspecting scatter and MA plots of probe-wise intensity before and after normalization. For all include experiments, differentially expressed genes were identified using the moderated t-statistic [82] implemented in the LIMMA R-package. Cross-species analysis using was performed using the proposed method where up- and down-regulated genes were tested separately using one-sided tests. The most significant p-value was then selected. The cross-species p-values were finally corrected for multiple testing using Benjamini-Hochbergs false discovery rate.

#### Heat stress

Gene expression data from eight experiments investigating the effects of heat stress in eight species were fetched from Gene Express Omnibus and ArrayExpress (Table 1). Homologene release 65 was used to describe the evolutionary relationship between the genes from the different species. The arbitrary component of the weights was set to be uniform over the eight experiments. The homology groups were populated with Gene Ontology terms based on species-specific annotations retrieved from the GO Consortium FTP (ftp://ftp.geneontology.org/pub/go/gene-associations/). Only terms with an experimental evidence code (i.e. EXP, IDA, IPI, IMP, IGI and IEP) were considered. Functional enrichment was inferred using the topGO R package [83].

#### Estrogen exposure

The five gene expression experiments included in the analysis are summarized in Table 2. Gene expression data was retrieved from the Gene Expression Omnibus, ArrayExpress or through direct contact with the authors. Homology groups were inferred from the corresponding EST and transcript sequences using OrthoMCL [41] with an inflation index of 1.5 (all other parameters had default values). To avoid bias from the multiple experiments performed in *Oncorhynchus mykiss* the arbitrary weight component was set to 0.25, 0.25, 0.25, 0.125 and 0.125 (following the order in Table 2).

## Competing interest

The authors declare that they have no competing interests.

## Authors’ contributions

EK, TÖ, LG planned the study. The method was developed by EK, TÖ and ON, implemented by EK and TÖ and evaluated by EK, LG, GA. EK and LG performed the meta-analysis of the heat stress microarray data. TÖ, LG and EK performed the analysis of the meta-analysis of estrogen exposure microarray data. EK, LG and TÖ wrote the paper. EK, ON and DGJL supervised the work. All authors read and approved the final manuscript.

## Supplementary Material

Additional file 1 **Additional figures demonstrating the power of the method using simulations.** Power characteristics for the proposed and previously suggested methods. The file contains results from the following simulations: (1) multiple in-paralogs with similar expression profile (2) multiple in-paralogs with divergent expression profile, (3) noise with thick tails (t-distribution with five degrees of freedom), (4) errors in homology structure, error rate=0.1 and (5) errors in the homology structure, error rate=0.5. Click here for file

Additional file 2 **List of analyzed homology groups from the meta-analysis of heat stress experiments.** Results for each homology group based on heat stress experiments performed in eight different species. The list contains combined p-value and false discovery rate as well as individual p-values from each experiment. Click here for file

Additional file 3 **Results of the functional enrichment of Gene Ontology terms.** Results from the Gene Ontology (GO) term enrichment analysis of the significant homology groups from the heat stress analysis. The file contains results from the biological process (BP), cellular component (CC) and molecular function (MF) ontologies. Click here for file

Additional file 4 **List of analyzed homology groups from the meta-analysis of aquatic vertebrates exposed to estrogens.** Results for each homology group based on estrogen exposure experiments performed in four aquatic vertebrates. The list contains combined p-value and false discovery rate as well as individual p-values from each experiment. Click here for file
