# Supplementary material for: A novel method for cross-species gene expression analysis
Source: BMC Bioinformatics. 2013 Feb 27;14:70. doi: 10.1186/1471-2105-14-70 (PMC3679856; doi:10.1186/1471-2105-14-70)
Supplement: Additional file 1 — Additional figures demonstrating the power of the method using simulations. Power characteristics for the proposed and previously suggested methods. The file contains results from the following simulations: (1) multiple in-paralogs with similar expression profile (2) multiple in-paralogs with divergent expression profile, (3) noise with thick tails (t-distribution with five degrees of freedom), (4) errors in homology structure, error rate=0.1 and (5) errors in the homology structure, error rate=0.5. [file 1471-2105-14-70-S1.pdf]

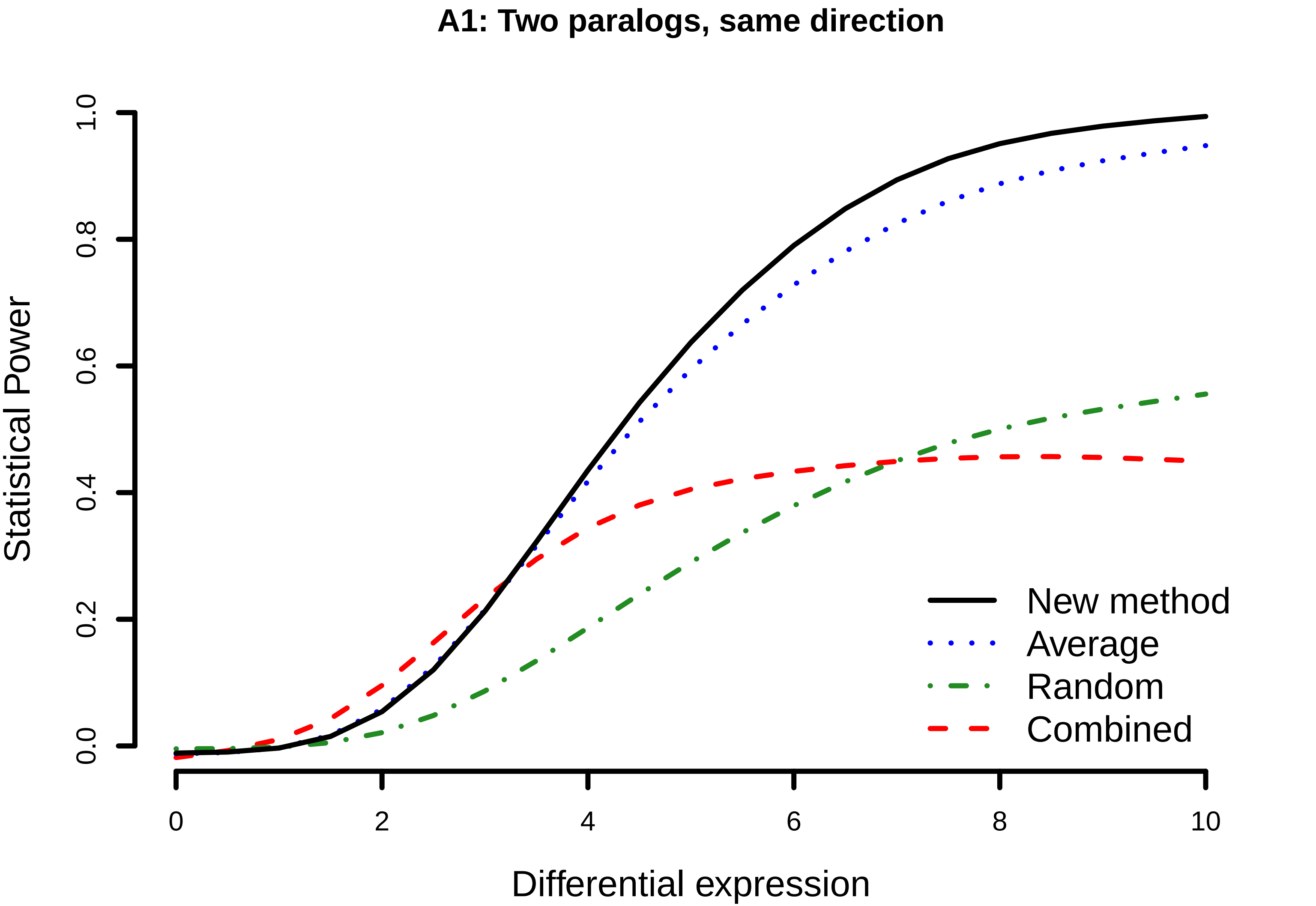

A1: Two paralogs, same direction
Statistical Power
0.0
0.2
0.4
0.6
0.8
1.0
0
2
4
6
8
10
Differential expression
New method
Average
Random
Combined

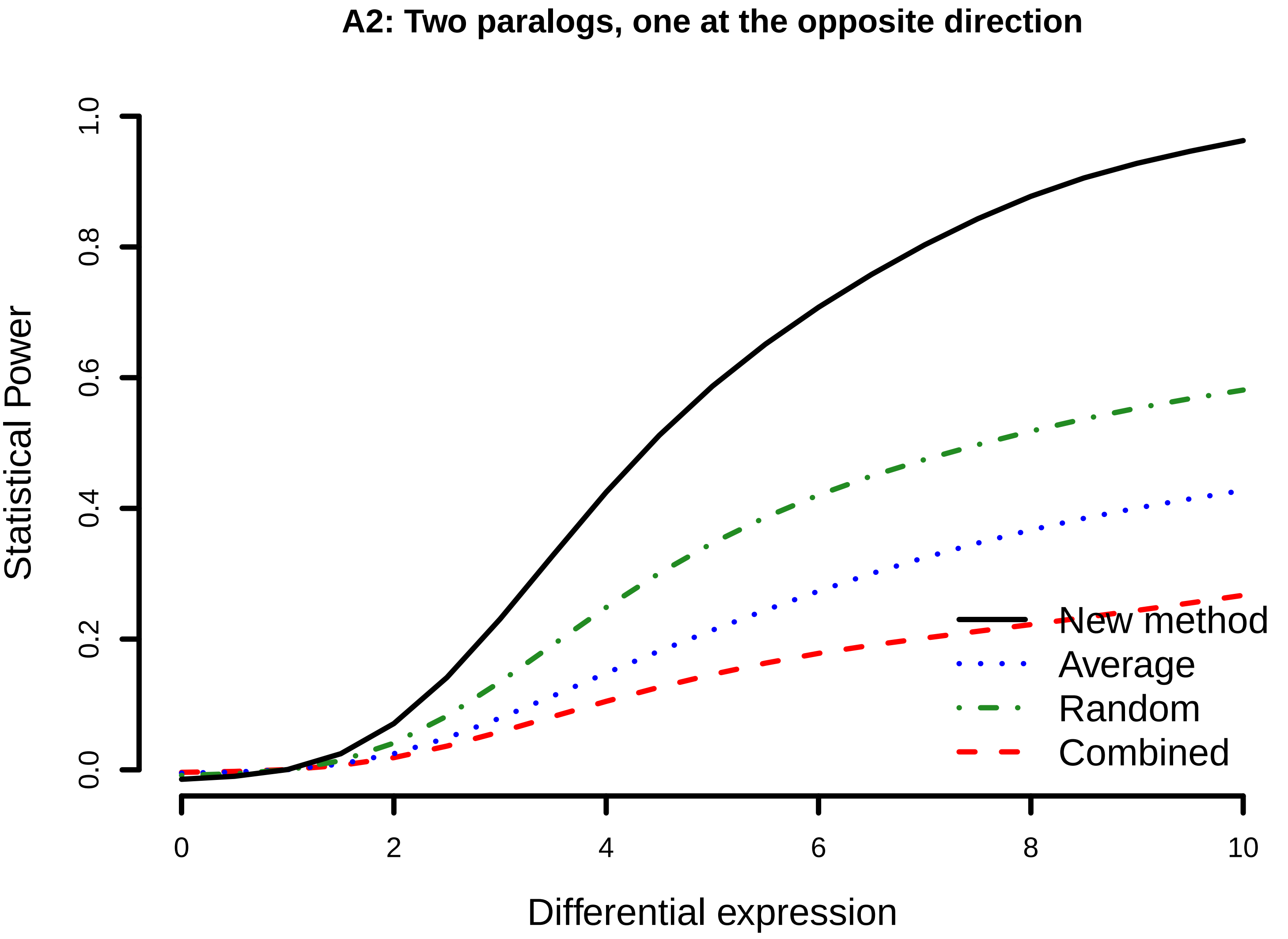

A2: Two paralogs, one at the opposite direction
Statistical Power
0.0
0.2
0.4
0.6
0.8
1.0
0
2
4
6
8
10
Differential expression
New method
Average
Random
Combined

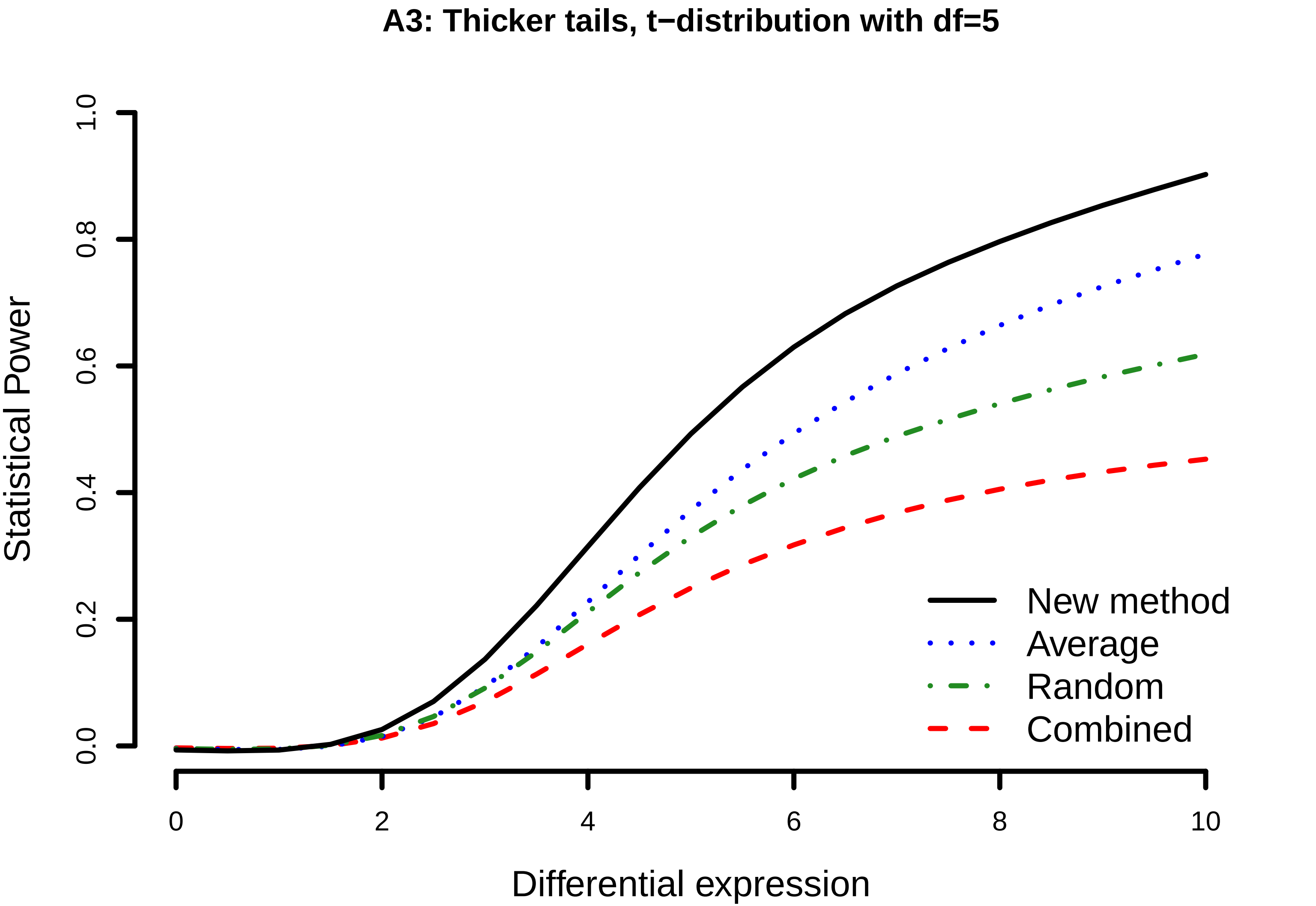
A3: Thicker tails, t−distribution with df=5
Statistical Power
0.0
0.2
0.4
0.6
0.8
1.0
0
2
4
6
8
10
Differential expression
New method
Average
Random
Combined

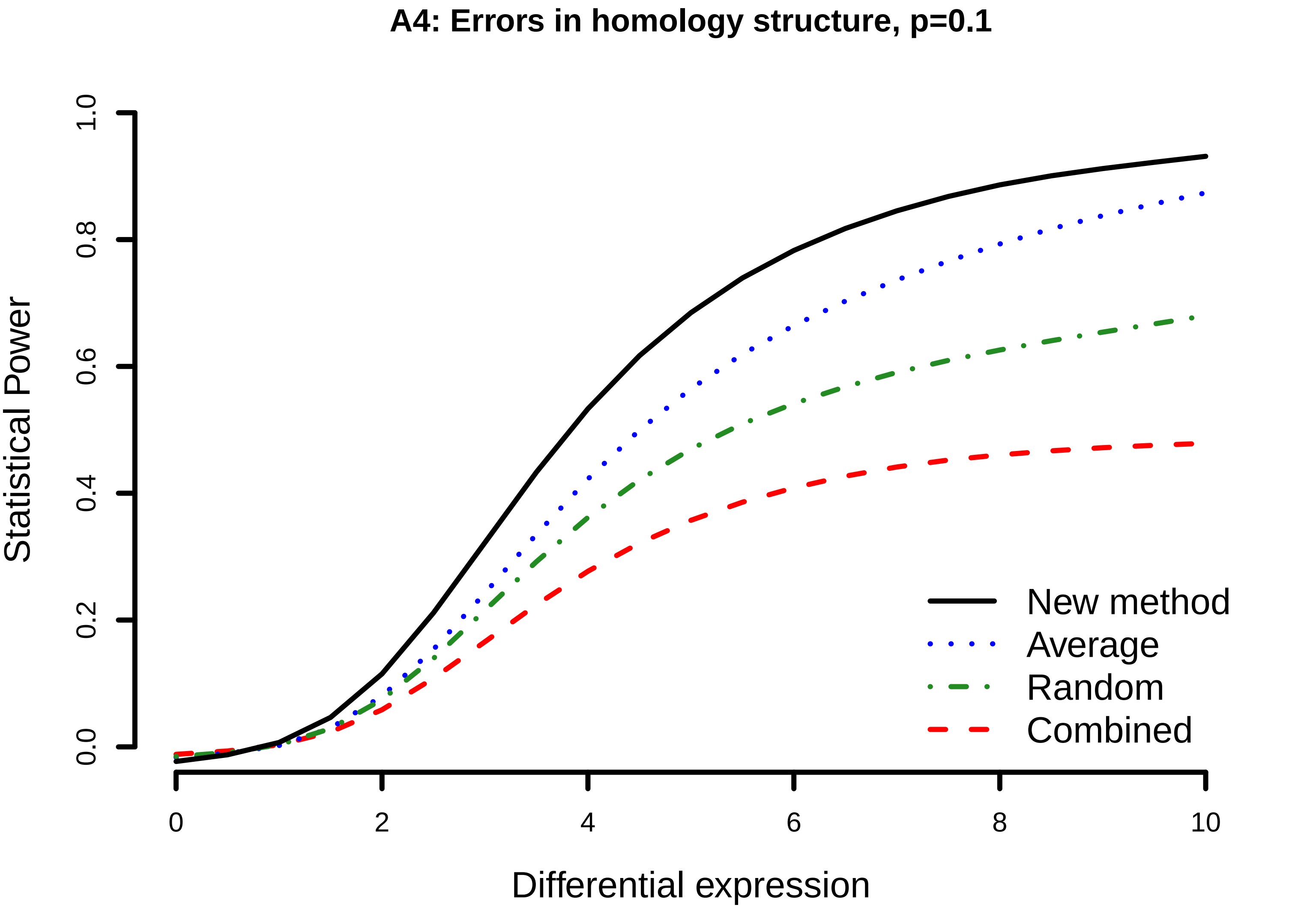
A4: Errors in homology structure, p=0.1
Statistical Power
0.0
0.2
0.4
0.6
0.8
1.0
0
2
4
6
8
10
Differential expression
New method
Average
Random
Combined

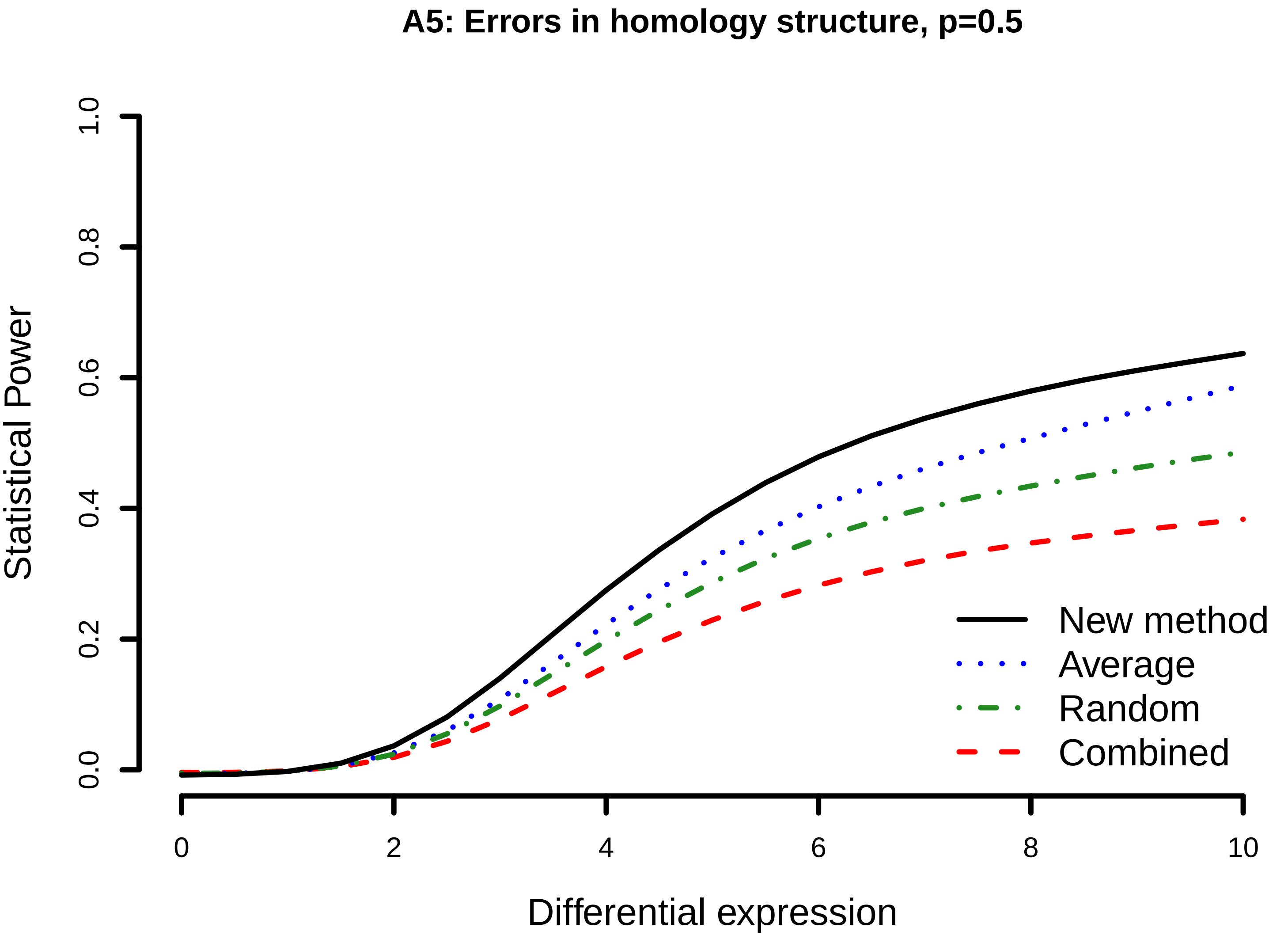
A5: Errors in homology structure, p=0.5
Statistical Power
0.0
0.2
0.4
0.6
0.8
1.0
0
2
4
6
8
10
Differential expression
New method
Average
Random
Combined
